# Cost-effectiveness of using a social franchise network to increase uptake of oral rehydration salts and zinc for childhood diarrhea in rural Myanmar

**DOI:** 10.1186/s12962-015-0030-3

**Published:** 2015-02-05

**Authors:** David Bishai, Karampreet Sachathep, Amnesty LeFevre, Hnin New Nwe Thant, Min Zaw, Tin Aung, Willi McFarland, Dominic Montagu, Social Franchising Research Team

**Affiliations:** Department of Population Family and Reproductive Health, Johns Hopkins Bloomberg School of Public Health, 615 N. Wolfe Street, Baltimore, MD 21205 USA; Department of International Health, Johns Hopkins Bloomberg School of Public Health, 615 N. Wolfe Street, Baltimore, MD 21205 USA; PSI-Myanmar, 16 West Shwe Gone Dine 4th Street, Bahan Township, Yangon, Myanmar; Department of Epidemiology, University of California San Francisco, San Francisco, CA USA

**Keywords:** Costing, Zinc, Oral rehydration, Private providers, Social franchise, Myanmar, Burma, Cluster randomized trial

## Abstract

**Introduction:**

This paper examines the cost-effectiveness of achieving increases in the use of oral rehydration solution and zinc supplementation in the management of acute diarrhea in children under 5 years through social franchising. The study uses cost and outcome data from an initiative by Population Services International (PSI) in 3 townships of Myanmar in 2010 to promote an ORS-Zinc product called ORASEL.

**Background:**

The objective of this study was to determine the incremental cost-effectiveness of a strategy to promote ORS-Z use through private sector franchising compared to standard government and private sector practices.

**Methods:**

Costing from a societal perspective included program, provider, and household costs for the 2010 calendar year. Program costs including ORASEL program launch, distribution, and administration costs were obtained through a retrospective review of financial records and key informant interviews with staff in the central Yangon office. Household out of pocket payments for diarrheal episodes were obtained from a household survey conducted in the study area and additional estimates of household income lost due to parental care-giving time for a sick child were estimated. Incremental cost-effectiveness relative to status quo conditions was calculated per child death and DALY averted in 2010. Health effects included deaths and DALYs averted; the former modeled based on coverage estimates from a household survey that were entered into the Lives Saved Tool (LiST). Uncertainty was modeled with Monte Carlo methods.

**Findings:**

Based on the model, the promotional strategy would translate to 2.85 (SD 0.29) deaths averted in a community population of 1 million where there would be 81,000 children under 5 expecting 48,373 cases of diarrhea. The incremental cost effectiveness of the franchised approach to improving ORASEL coverage is estimated at a median $5,955 (IQR: $3437-$7589) per death averted and $214 (IQR: $127-$287) per discounted DALY averted.

**Interpretation:**

Investing in developing a network of private sector providers and keeping them stocked with ORS-Z as is done in a social franchise can be a highly cost-effective in terms of dollars per DALY averted.

**Electronic supplementary material:**

The online version of this article (doi:10.1186/s12962-015-0030-3) contains supplementary material, which is available to authorized users.

## Background

Diarrheal diseases remain the second leading cause of death amongst children under 5, accounting for an estimated 0.75 million deaths globally [1]. An estimated one-third of all global child deaths occur in Southeast Asia in 2010 [1]. Emerging from decades of political and economic isolation, Myanmar reports some of the highest child mortality rates in the region. Diarrheal disease along with acute respiratory infections and malaria exacerbated by underlying malnutrition continue to be the main direct causes of deaths among children under 5 in the country [2,3]. Diarrhea is the second leading cause of child death in Myanmar, causing 13% of deaths of children under five [2].

Zinc supplementation for 10–14 days in conjunction with ORS has been demonstrated to significantly lesson the duration and severity of diarrheal episodes while reducing reoccurrence in the ensuing 2–3 months [4-6]. In response to empirical evidence demonstrating the impact of therapeutic supplementation of zinc and ORS (ORS-Z) for the management of acute diarrhea on health outcomes, including mortality, UNICEF and WHO issued a joint statement in 2004 recommending a change in global guidelines for the management of acute diarrhea to include supplementation with 20 mg of disbursable zinc sulphate in addition to reduced osmolarity ORS. Following these guideline changes in 2004, there have been several efforts to ensure that children with diarrhea in poor countries actually receive adequate doses of zinc and ORS. Significant challenges remain in establishing systems for promoting high coverage especially in rural areas [4-6].

In Myanmar, careseeking for diarrhea treatment is limited [7]. In rural areas, where 70% of Myanmar’s population reside, 40% of individuals that do seek care for diarrhea seek care first from private providers [2]. Given population preferences for careseeking among this sector, any strategy to expand coverage of zinc and ORS would either need to increase product prescription in private facilities or find a way to switch treatment seeking to government facilities [2].

Social franchising offers a viable strategy to increase the uptake of health commodities in private facilities, but it can be costly to implement [8]. In a social franchise, an NGO forms a network of private providers who agree to regular visits and distribution of services and commodities. The NGO will obtain financing to support its distribution network and to offer subsidies for the commodities. The costs of forming and maintaining the network and applying subsidies can be 2–3 times as much as the direct cost of the commodities [8]. However without the NGO’s investments, the private sector may not achieve adequate distribution of commodities. This study measures the cost per DALY averted by achieving increased coverage of ORS-Z using a social franchise. The comparator, baseline condition is to continue making ORS and zinc available in government clinics, and to do nothing to promote their sale in private retail outlets.

Population Services International (PSI) Myanmar has developed ties to over 1000 private sector providers though a social franchising program that was initially focused on reproductive health. In the last 10 years disease treatment lines for malaria and STIs (2003), tuberculosis (2004), and diarrhea and pneumonia in children under 5 (2008) have been rolled out. There are two separate networks in PSI Myanmar: Sun Quality Health (SQH) network includes mostly physicians and advanced health professionals while Sun Primary Health (SPH) providers typically have a lower level of training and often operate as mobile providers in an effort to reach those most at risk and in rural areas. In the franchised delivery model “Sun Field Leaders” serve as local supervisors who link Sun Primary Health providers in the private sector to PSI/Myanmar quality improvement and product development support. The financing for the PSI provision of quality improvement comes from outside donations, typically from development assistance.

The cost effectiveness analysis was conducted in the context of data on program impact from a community-level, cluster-randomized trial of the introduction of ORASEL product through a social franchising program. The effect of the social franchising program on changes in the use of ORASEL was measured with before and after household surveys in a quasi-experimental community randomized trial that was conducted in 2010.

## Methods

Oral rehydration salts and zinc are individual treatments and the effects from treating one patient are well known [5,6,9]. However the subject of this paper is neither the effectiveness nor the cost-effectiveness of the clinical treatment of one patient with ORS and zinc. Our focus is to evaluate a public health intervention designed to promote uptake of ORS-Z in a community.

A study of a clinical treatment could naturally be evaluated on the basis of clinical effects achieved per patient treated. However a study of a public health intervention needs to be evaluated on the basis of a standard size community treated. In *Disease Control Priorities* 2^nd^ Edition the standard size community was typically 1 million. So for comparative purposes we normalize our evaluation to estimate total costs and total lives saved per 1 million total population in a community that chose to promote more uptake of ORS-Z using the strategies under consideration.

Myanmar’s population is 53 million of whom 8.1% are under age 5 [10]. The incidence of diarrhea in children under 5 in Myanmar is 59.7 cases per 100 person years [11]. Given Myanmar’s demography, a population of 1 million would have 81,000 children under 5 [10]. Given Myanmar’s epidemiology, a population of 81,000 children would have 48,373 cases of diarrhea per year [10].

### Intervention and setting

The intervention is adding ORS-Z as an additional product line in an existing social franchise program. Note that this is not an economic evaluation of ORS-Z as a product. It is an economic evaluation of social franchising as a platform that might enable cost-effective distribution of products such as ORS-Z.

The study generating data on costs and program effects was conducted in three townships of Myanmar; one in the south (Wakema) and two in the north (Tada-u and Myittha) with an estimated cumulative total population of 190,000. The study area comprised 104 village tracts in three rural townships of Myanmar, matched in 52 comparable randomly assigned pairs to either the intervention of PSI’s system of distributing ORASEL at commercial venues and enrolling SQH workers to provide ORASEL in the community or a control condition where PSI did not intervene and the availability of zinc and ORS was determined by local government policy and local market forces. More detail on the exact nature of the intervention is available in the Aung et al. paper [11].

The PSI ORASEL KIT® contains two sachets of low-osmolarity oral rehydration salts (ORS) and one course of zinc treatment (10 tablets of 20 mg), in accordance with WHO and UNICEF recommendations and protocol for the treatment of diarrhea in children under the age of five (UNICEF stipulates a 10–14 day regimen) [9]. The ORASEL product was developed by PSI-Myanmar and the country office had to source the ingredients, develop the packaging and obtain government clearance prior to product launch. Health workers and drug vendors sell the ORASEL kits to households at a low, subsidized price equivalent to $0.35 per kit.

### Estimating health outcomes

DALYs were calculated to facilitate the comparison of cost-effectiveness across alternative resource uses. Deaths averted were modeled using the estimated increment in coverage for ORASEL attributable to the intervention. The best approach to estimating the coverage increment was to use a difference in difference estimate that compared the change in ORASEL use in the intervention area to the change in ORASEL use in the matched comparison area.

This coverage increment was fed into the Lives Saved Tool (LiST) to estimate an increment in lives saved based on parameters customized to rural Myanmar. Years of life lost by children due to disability from acute diarrhea were deemed negligible compared to years of life lost due to death so the DALYs in this model are all due to years of life lost due to premature death [12]. This is a conservative estimate that will under-estimate the benefits of ORS-Z by leaving out the years of life lost due to acute disability from diarrhea.

### Calculating costs

**Figure 1:**
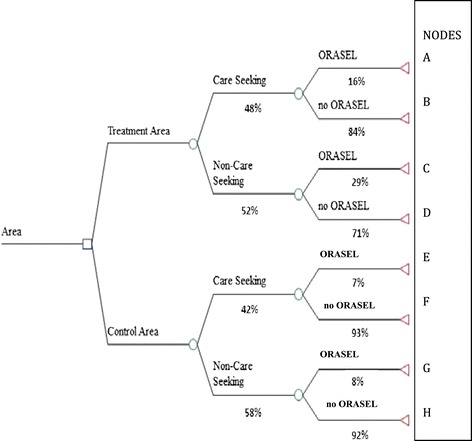
**The decision tree used to model the expected survival rates for the null intervention (control) area and the treatment area, with baseline probabilities.** For each end-point, patients either survive or die depending on the probabilities reported in Table 1.

**Figure 2:**
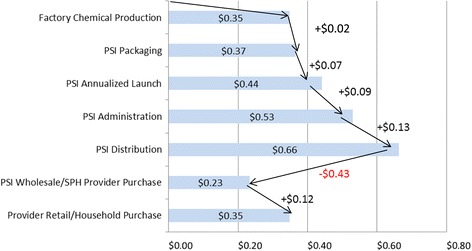
**ORASEL value chain per packet.** Blue bars show cumulative value of the product at each point in production. Rightward arrows all add value to the product. Leftward arrow shows that PSI subsidizes the product by $0.43 to promote distribution. Retail services by SPH providers add $0.12. Total value of all rightward arrows is the social cost of ORASEL and comes to $0.66 + $0.12 = $0.78.

Economic costs were estimated from the medical and societal sector perspective using an ingredients approach. A simple decision tree for children with diarrhea separated the probability of seeking any formal treatment from the probability of receiving ORS-Z conditional on either being seen by a health provider or being cared for at home. The tree had 4 terminal nodes for the intervention area and 4 comparable terminal nodes for the control area (Figure 1). The expected costs at each terminal node depended on adding up the unit cost of a provider visit and the unit cost of a treatment with ORASEL times the respective probabilities. Since ORASEL and its distribution in the intervention area were heavily subsidized, it would not be accurate to simply apply a retail price to each unit of ORASEL. Extensive data were collected from PSI personnel on the resources that would be necessary to make a product like ORASEL become available and adopted for use among sick children in rural Myanmar. Figure 2 tracks the value added by PSI from factory gate to retail purchase, and identifies a total cost of $0.78 per unit. PSI uses donor funds to finance a $0.43 per unit subsidy to achieve a retail price of $0.35, but the subsidy does not negate the cost.

Data came from a retrospective review of financial reports maintained by PSI-Myanmar’s Finance Department in Yangon. Key informant interviews with staff in the central Yangon office were also conducted in order to better understand organizational and product divisions and emphasis was placed upon the identification of PSI payroll and cost data as well as determining the percentage of franchise time spent on delivering ORASEL.

This analysis takes a societal perspective on costs. Costs are accrued by the franchisor (PSI), the franchisee (Private provider), and by the patient. These may be summarized as follows: 1. Costs to PSI headquarters for program launch and product acquisition; 2. Costs to PSI associated with the packaging, distribution, and service monitoring related to ORASEL products channeled through the franchise network; 3. Costs to households associated with loss of wages for treating diarrhea and care-seeking; 4. Costs to providers for stocking and selling the ORASEL product at retail; 5. Costs of providers in taking time and effort to deliver professional services in assessing sick children and recommending treatment. The revenue to finance all these costs ultimately comes from both patients who pay to purchase services and commodities and from outside donors who support PSI’s efforts in product launch, distribution, and the administration of commodity subsidies. All costs and monetary values are presented in 2010 dollars (US$). Costs in Burmese Kyat in years prior to 2010 were inflated using local consumer price indices obtained from the IMF’s International Financial Statistics Yearbook, then converted to US dollars using Myanmar’s currency exchange rate. The time horizon is one year for both costs and health outcomes. This horizon is appropriate assuming fixed costs of launching the intervention have been annualized. The duration of diarrhea is far less than one year, but budgeting is typically annual.

### Program costs components

#### Product launch costs

Several activities were necessary at PSI headquarters before ORASEL could be distributed in Myanmar. These pre-launch costs included planning meetings to assess the audience and product profiles, and to design packaging and promotional material and plan a marketing campaign. Through key informant interviews we were able to document a thorough list of the pre-launch and launch activities and to estimate the total sunk costs. We assumed that these sunk costs would have straight-line depreciation for 10 years and we apportion these sunk costs to each unit of ORASEL that PSI anticipated selling over the 10 year period.

#### Costs associated with the packaging and distribution of commodities

Once procured at internationally competitive prices, commodities are packaged in Yangon-based warehouses by PSI staff. The packaging was designed by PSI’s marketing and communications department and produced locally in Myanmar. Financial records were reviewed to determine costs associated with the packaging, re-sale and distribution of products in Myanmar. Product distribution to clinics in PSI’s network includes supervision of service quality during monthly visits to each member. PSI/Myanmar’s franchise officers are all physicians and they spend several hours during each visit reviewing clinical records, and discussing case management with providers.

Coordinating distribution incurs overhead costs at headquarters, many of which are shared costs. There are two levels of shared costs: 1) The share of ORASEL in all of the cost of the franchise program; 2) The share of the franchise program in all of the work that PSI does in Myanmar. To allocate shared overhead costs to the ORASEL program within the franchise program we identified the cost of all commodities distributed in the franchise program and calculated ORASEL’s share in these costs. The share of franchising in all of the work of PSI was allocated at approximately 33%. This percentage reflects the total proportion of PSI’s annual franchising budget out of the total annual operating budget for all programs. Direct costs of distributing ORASEL associated with transportation, staff salaries, program incentives, etc. were obtained through an intensive review of PSI’s financial records and cross-checked through in-depth interviews with PSI staff to ensure the exhaustive inclusion of all potential expenditures.

#### Costs incurred by providers in the delivery of commodities and services

Providers in PSI’s network agree to a PSI fee schedule in charging patients for the commodities and professional services that are required to accompany the PSI commodities. With data on the number of visits we used the fee schedule to calculate the amount of revenue for providers for the medical services related to diarrhea. Monthly visits by PSI staff are used to assure that providers publicly post a price list. A household survey in 2010 asked households in both control and intervention areas how frequently they sought care for diarrhea in their children and what they paid to access diarrhea care in non-PSI providers. Guided by the decision tree in Figure 1, we used this information to estimate the expected costs of diarrhea for households in the treatment area and households in the control area [8].

#### Parent’s lost time

From the societal perspective a sick child represents an opportunity cost in the family’s allocation of time. A caregiver such as a mother, sibling, or father will reallocate time from leisure or productive employment to attend to the sick child. This time reallocation occurs whether the caregiver is primarily engaged in formal sector employment, subsistence farming, schooling, or unpaid domestic work. To place a locally relevant monetary value on the reallocation of time we used GDP/capita/day in Myanmar as of 2010 to proxy the value of time that had to be taken from other activities by a caregiver for every day a child had diarrhea. We assumed that ORASEL could reduce the duration of an average episode of diarrhea by one day, assuming perfect adherence [5]. In sensitivity analysis we assessed the impact of assuming that parents’s lost time had zero value—this is equivalent to the medical sector model.

### Intervention effectiveness

We base our analysis on a single effectiveness study that evaluated the impact of introducing ORS-Z into a social franchise. The literature evaluating the impact of social franchising on health is small and the types of health interventions is too heterogeneous to support a synthesis approach. The effectiveness study that we use was a quasi-experimental community trial that was able to measure the difference in ORS-Z use that could be attributed to PSI’s social marketing efforts using difference in difference analysis of pre-post differences between the intervention area and the control area from 2010–2011 [11]. A household survey measured the incidence of diarrheal episodes in children in both comparison and intervention areas. The analysis estimated the average zinc and ORS use rates for children with diarrhea in 52 pairs of matched control and intervention villages before and after the intervention.

The Lives Saved Tool (LiST) is a validated computer program developed by the Child Health Epidemiology Research Group that uses regularly updated parameters about public health program impact and the current local epidemiology to estimate how incremental changes in coverage for critical maternal, newborn and child health services translate into the number of lives saved. Findings emerging from household survey data were input into LiST to estimate the number of lives saved among children under 5 due to diarrhea as a function of the increase in coverage of zinc and ORS [12].

Estimates of lives saved were translated into Disability Adjusted Life Years (DALYs) – a summary measure of disease inclusive of both morbidity and mortality. DALYs were calculated using a 3% discount rate as recommended by the WHO for analysis of diarrhea control programs [13-15]. There was no age weighting, and we assumed children would achieve the current average life expectancy at birth of 65 for Myanmar [16]. Age at death was equal to the mean age of patients reported as having a diarrhea episode at the time of the household survey.

The analysis derived parameters on how social franchising impacts coverage with zinc from a sub-national study in rural Myanmar. It combined these with LiST tool estimates that translated ORS-Zinc coverage into estimates of lives saved based on demography and child mortality that applied at the national level for Myanmar. Our analysis relies on LiST tool’s model of how well ORS-Z would work in Myanmar. These are important assumptions, made necessary because it would have been too costly and inefficient to power a trial of social franchising to an endpoint of mortality.

### Uncertainty and sensitivity analysis

A second order Monte Carlo simulation was conducted on cost and impact parameters in the decision tree. The cost parameters were modeled with triangular distributions with minimum and maximum values at +/− 20% of the baseline value. The impact parameters measuring the probability of seeking care and the probability of receiving ORS-Z if care was sought were modeled as normal distributions with mean equal to the observed proportion seeking care based on the household survey and standard deviation calculated as SD = p(1-p) × N^1/2^. Interquartile ranges were used to communicate an uncertainty range for resulting incremental cost effectiveness ratios.

## Results

### Health impact

The ORASEL impact study produced a difference in difference estimate showing that ORS plus zinc use increased significantly in the intervention tracts from 6.1% (0.3%-12%) to 13.7% (7.3%-20.2%), as compared to slight statistically insignificant declines in ORS use in the comparison area (4.8% to 1.8%) over the same time period [11]. Since there was no increase in zinc and ORS use in the matched control villages, we can identify the treatment effect as a 7.6% increase in zinc and ORS use which is the pre-post difference in the intervention area. This is an optimistic estimate of the impact of PSI on ORS-Z uptake. Among those who received the ORS-Z, adherence was not measured, and we must assume it was as good as adherence in the original ORS-Z studies that informed the LiST tool. Based on LiST, an increment of 7.6% in the use of ORS-Z, would translate to 2.85 (SD = 0.29) incremental deaths averted in a total community population of 1 million.

### Incremental costs from the societal perspective and medical perspective

Figure 2 displays the ORASEL value added chain per ORASEL kit. The total cost to society for an ORASEL kit is 78 cents. Chemical production costs are estimated at 35 cents per kit. PSI then pays 2 cents per unit to package these ingredients in its warehouse. The annualized costs incurred to launch PSI applied to the projected sales of ORASEL for 10 years come to $0.07 per unit. Overhead attributable to the ORASEL product comes to $0.09 per unit and the distribution costs are $0.13 per unit. The sum of all these investment is $0.66, then PSI marks the wholesale price down to $0.23 to sell it to its network of private health providers. The suggested retail price is $0.12 higher at $0.35. Ultimately the household pays $0.35 per unit and PSI and its donors pay $0.43 per unit.

As noted in the decision tree, some households obtain ORS-Z after a sick child visit for diarrhea and some obtain it from a pharmacy or drug shop without seeing a health provider. The social franchising intervention worked with PSI’s informal sector providers and was found to increase the odds of receiving ORS-Z in venues other than primary care. A process of folding back the tree diagram (Figure 1) was used to calculate the estimated costs of each terminal node representing the pathway taken to respond to a child with diarrhea. Table 1 shows the unit costs used in the model. Table 2 shows the subtotals of the costs incurred at the various nodes. For instance one child arriving at node A requires provider costs ($2.00) and a dose of ORASEL ($0.78). One child arriving at Node B requires provider costs ($2.00) and because they do not get ORASEL the household members suffer an extra day of having a child with diarrhea for an additional $1.35. The “per diarrhea” columns of Table 2 multiplies the terminal node cost per event totals times the relevant probabilities that one patient with diarrhea would end up at each terminal node in the tree. The estimated societal cost per diarrhea of a child in the intervention area would be the sum of $0.21 + $1.34 + $0.11 + $0.50 = $2.16. The expected cost per diarrhea in the control area would be the sum of $0.05 + $1.09+ $0.01 + $0.68 = $1.83. The higher expenses in the treatment area are due to a higher probability of using ORASEL. This higher cost amounts to an extra $0.33 per case of diarrhea. One static estimate of total costs and incremental costs is shown in Table 2, but a stochastic estimate of these costs is shown in Table 3. As per Table 3, in a population of 1 million people expected to have 81,000 children under 5 and 48,373 cases of diarrhea the cost of diarrhea treatment would be $105,858 (SD $6067) in a population receiving a socially franchised ORASEL program and $89,386 (SD 5352) with ordinary government care. The incremental cost of the program from the societal perspective would be $16,472 (SD 8,494).

**Table 1:** **Unit costs**

**Unit cost**	**Unit cost**	**Range for sensitivity analysis**
Factory chemical production	$0.35	+/− 20%
Packaging	$0.02	+/− 20%
Launch	$0.07	+/− 20%
Cost of PSI administration	$0.09	+/− 20%
Franchise Officer ORASEL Distribution by PSI	$0.13	+/− 20%
ORASEL retail cost by SPH provider	$0.12	+/− 20%
Subtotal program cost per ORASEL user	$0.78	+/− 20%
Household costs Expected value of hospitalizations per diarrhea if no ORS	$0.07	+/− 20%
Expected value of mothers lost productivity if no ORS	$1.28	+/− 20%
Subtotal household costs if ORASEL not used	$1.35	+/− 20%
Medical Provider Costs Treatment area consultation cost for diarrhea episode	$2.00	+/− 20%
Control area consultation cost for diarrhea episode	$1.50	+/− 20%

Data from Key Informants in PSI.

**Table 2:** **Costs and incremental cost**

**Societal perspective**	**Intervention area**		**Control area**		**Incremental cost per diarrhea**
	**Cost per event**	**Cost per diarrhea**	**Cost per event**	**Cost per diarrhea**	
Seeks care & gets ORASEL [a]	$2.78	$0.21	$1.85	$0.05	$0.16
Seeks care & no ORASEL [b]	$3.35	$1.34	$2.78	$1.09	$0.25
Doesn't seek care & gets ORASEL [c]	$0.78	$0.11	$0.35	$0.01	$0.10
No care, no ORASEL [d]	$1.35	$0.50	$1.28	$0.68	$0.18
Estimated cost per diarrhea		$2.16		$1.83	$0.33
Total estimated cost for 48,373 cases		$104,486		$88,522	$15,963

Costs per event rationale as follows.

[a]$0.78 for ORASEL and $2.00 for consultation=$2.78 or $0.35 for ORS-Zinc plus $1.50=$1.85.

[b] $2.00 for care seeking plus $1.35 for household cost =$3.35 or $1.50 for care seeking plus $1.28 for household cost=$2.78.

[c] $0.78 for ORASEL or $0.35 for ORS-Zinc.

[d] 1.35 for household costs or $1.28 for household costs.

**Table 3:** **Costs, Effects, and incremental cost-effectiveness emerging from Monte Carlo model of costs and program effects**

	**Total costs**		**Incremental effects (Intervention minus control)**		**Incremental cost**	**Incremental cost per DALY averted**	**Incremental cost per Death averted**
	**Intervention**	**Control**	**DALY’S averted**	**Deaths averted**			
Avg from 1000 iterations	$105,858	$89,386	77.28	2.85	$16,472	$214	$5,744
SD	(6,067)	(5,352)	(7.94)	(0.29)	(8,494)	(111)	(3,058)
Median						$214	$5,955
Lower 25%						$127	$3,437
Upper 25%						$287	$7,589
Base case from tree	$104,486	$88,523	65.63	2.43	$15,963	$243	$6,582

The bottom row labeled base case from tree offers the results from a non-stochastic estimate. The non-stochastic estimate lies close to both the mean and the median of the stochastic estimates. The stochastic incremental cost average estimate of $16,472 (SD $8,494) which emerged from 1000 iterations, lies close to the non-stochastic estimate of $15,963 shown in Table 2.

From the medical perspective the incremental costs of the program are higher because the medical perspective does not include the saved value of the mother’s lost productivity due to more disability days of diarrhea. From the medical perspective the incremental cost of the program would be $25,736 (SD $5655) because the mother’s lost productivity due to preventable diarrhea morbidity is estimated at $9,264 in the population of 1 million people (with 48,373 cases of childhood diarrhea).

### Incremental effects

Probabilities displayed in Figure 1 are the estimates of care seeking patterns and ORS-Z use at the individual level based on household survey data from 6582 households (3200 intervention and 3382 control). The probabilities are based on the frequencies in individual children and are not an average of village averages. We applied Bayes’ rule to calculate the probability of getting any ORS-Z as

$$ \mathrm{P}\mathrm{R}\left(\mathrm{Any}\ \mathrm{O}\mathrm{R}\mathrm{S}\hbox{-} \mathrm{Z}\right) = \kern0.37em \left[ \Pr \left(\mathrm{Seen}\right)\kern0.37em \times \Pr \left(\mathrm{O}\mathrm{R}\mathrm{S}\ \mathrm{if}\ \mathrm{S}\mathrm{een}\right)\right] + \kern0.37em \left[ \Pr \left(\mathrm{Not}\ \mathrm{S}\mathrm{een}\right) \times \Pr \left(\mathrm{O}\mathrm{R}\mathrm{S}\ \mathrm{if}\ \mathrm{not}\ \mathrm{seen}\right)\right] $$

(Statistical details and confidence intervals for each probability shown in Figure 1 are available from the authors upon request.)

### Incremental cost effectiveness

Table 3 shows the incremental costs, effects and cost-effectiveness. The first row shows the average from 1000 iterations and the third row shows the standard deviation of these 1000 iterations. The median incremental cost per child death averted is $5955 (IQR: $3437-$7589) and the median incremental cost per DALY averted is $214 (IQR: $127-$287). The last row shows the results when mean values are used to do the arithmetic.

The medical sector perspective is not shown in Table 3, but because the incremental costs appear higher from the medical perspective the cost effectiveness is less attractive from the medical perspective. The median incremental cost per child death averted from the medical perspective is $8980 (IQR: $7468-$10,391). The median incremental cost per DALY averted from the medical perspective is $339 (IQR: $276-$384).

### Sensitivity analysis

**Figure 3:**
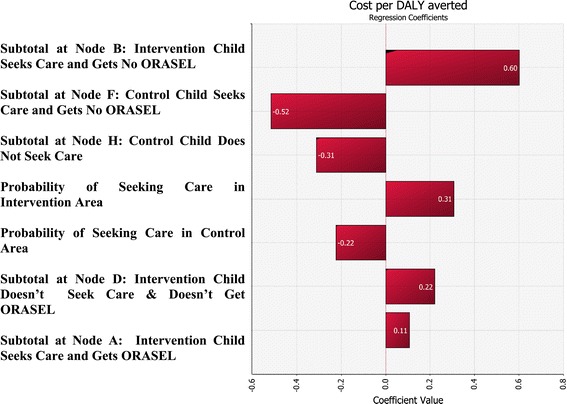
**Tornado diagram of univariate sensitivity analysis.** The 7 most influential variables are shown.

**Figure 4:**
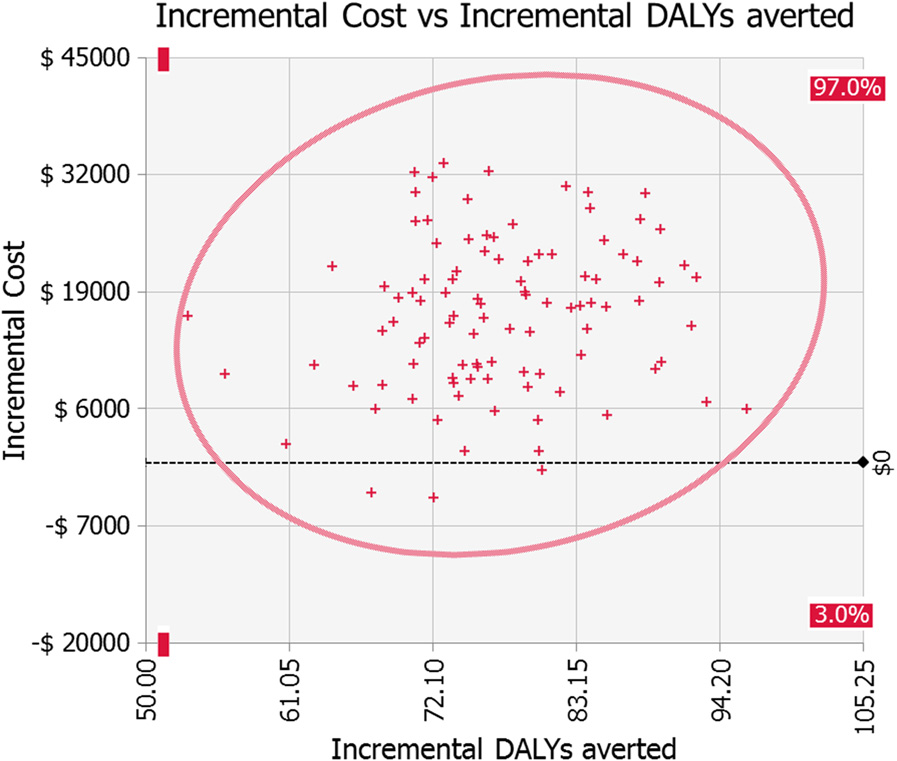
**Scatter plot of costs vs. effects.** Only 2% of iterations showed incremental cost less than 0. No iterations showed an incremental cost higher than $45,000 in a population of 1 million Myanmar total population.

Comparing the ICERs of the medical and societal perspective indicates how sensitive the results are to the assumption that mother’s lost time is valued highly. The assumption that mother’s time has zero value worsens the ICER for DALYs by about 30%, increasing it from $214 to $339. Figure 3 shows which variables are most influential for the cost per DALY averted based on regression analysis of the input data and output data from 1000 iterations. Figure 3 reveals that the incremental cost-effectiveness ratio was most sensitive to the probability that a child will get ORASEL if they seek care. The second most important parameter was the probability of care seeking for diarrhea. Figure 4 shows a scatter plot of the output from 1000 iterations showing that in only 3 out of 1000 iterations the intervention is cost saving.

## Discussion

Distribution of ORASEL through private sector franchising is a cost-effective investment. The extra cost incurred by PSI to distribute and market ORASEL in the private sector leads to an increment in coverage that saves enough lives to justify the investment. According to the thresholds for cost effectiveness set forth by the Commission for Macroeconomics and Health, the cost per DALY of an intervention needs to be below the GDP per capita of a country to be considered highly cost-effective. The Myanmar GDP per capita in 2010 was US$ 876.2 (2010 estimate) [13]. The franchising based distribution of ORASEL kits produces an incremental cost per DALY averted of $214 (IQR: $127-$287), from a societal perspective and $339 (IQR: $276-$384) from the medical perspective. Thus conventional criteria indicate that delivery of ORASEL through a social franchise may be regarded as cost-effective [14].

It is important to remember that our cost effectiveness estimate applies to a population-based method for promoting better uptake of zinc and ORS in a population of 1 million people compared to standard practice. Our results should not be compared with a cost-effectiveness estimate of treating one child with zinc and ORS vs. one child not using zinc and ORS. Our estimate is designed for use by a public health planner considering delivery strategies for promoting zinc and ORS to improve the health of a population. The “one-child” estimate would be appropriately used by a clinician deciding how to efficiently treat their next patient with diarrhea.

A prior study of the cost-effectiveness of diarrhea treatment in one child estimated that therapeutic supplementation of zinc and ORS corresponded to a cost per DALY averted within the range of $13-$66 compared to treating a child with diarrhea with ORS [17]. Like many studies of the cost effectiveness of a medical treatment, there is limited applicability of this result to planning policy for a large population. Trying to get large numbers of children to use ORS and zinc for diarrhea is costly. The money required to get higher coverage is not included in the estimate of a $13 incremental cost effectiveness ratio (ICER). Our results are thus quite unique and a significant contribution to the field as we included costs spent in packaging, marketing, and distributing zinc and ORS and used a population perspective in assessing the program outcome.

The advantage of the franchise network in achieving efficient distribution of a commodity like zinc and ORS is that the health care providers were already in the community, supported by the fees they charged patients for professional services. They received a highly subsidized product, ORS-Z, and were given the incentive to distribute it in the form of a $0.12 markup per packet.

This analysis may not be widely generalizable because PSI-Myanmar was fortunate to have a large provider network and donor support for the subsidies for ORS-Z. However, the problem of getting commodity distribution to scale up efficiently is a very common problem in public health and few studies examine the economics of scaling up, making this an important addition to the evidence on large scale public health initiatives.

### Assumptions and limitations

The analysis is based on several assumptions. We assumed that one kit – including a 10 day course of zinc and 2 sachets of ORS – would be used to treat a diarrheal episode—with an adherence rate at least as good as the adherence in prior efficacy studies. We also made an assumption about attributing PSI’s overhead costs to ORASEL based on product volume forecasts from 2010. Further, this study considered most of the direct costs and effects of the social franchising mechanism but not necessarily all the indirect costs and effects. Although this is a CEA of a population in a specific geographic area, we have taken national averages when estimating number of franchising officers, supervisors, etc., and we assumed that these averages will apply because site specific data were not available.

These considerations aside, the current coverage estimates may underestimate the true impact on health given that they do not consider the potential protective effect of zinc in reducing the probability of recurrent episodes of diarrheal in the ensuing 2–3 months nor any secondary impact in reducing the incidence of acute lower respiratory infections.

## Conclusions

Social franchising to increase coverage with ORS-Zinc for the treatment of diarrhea in children under 5 years of age is a highly cost-effective intervention. While the clinical efficacy of therapeutic supplementation of zinc and ORS for the management of acute diarrhea in children under 5 is known, this is the first study to focus on the cost-effectiveness of achieving coverage increments through social franchising at the population level. We found that public investments can support product marketing, subsidization, and distribution efforts that increase coverage enough to save lives and remain cost-effective.
